# Lipid–Retinoid Metabolic Network Links Genetic Variation to Molecular Heterogeneity in Inherited Retinal Degeneration

**DOI:** 10.3390/ijms27156888

**Published:** 2026-08-01

**Authors:** Przemysław Sołek, Ewelina Cholewińska, Robert Rejdak, Katarzyna Nowomiejska, Katarzyna Ognik

**Affiliations:** 1 Department of Biochemistry and Toxicology, University of Life Sciences in Lublin, Akademicka 13, 20-950 Lublin, Poland; ewelina.cholewinska@up.edu.pl (E.C.); katarzyna.ognik@up.edu.pl (K.O.); 2 Department of General and Pediatric Ophthalmology, Medical University of Lublin, Chmielna 1, 20-079 Lublin, Poland; robert.rejdak@umlub.pl (R.R.); katarzyna.nowomiejska@umlub.edu.pl (K.N.)

**Keywords:** inherited retinal degeneration, lipid–retinoid metabolism, lipoprotein-associated pathways, metabolic networks, molecular remodeling, genetic variation

## Abstract

Inherited retinal degeneration (IRD) comprises a genetically heterogeneous group of disorders in which the mechanisms linking genetic variation to molecular heterogeneity remain poorly understood. Here, we investigated the contribution of lipid and retinoid metabolism to the molecular etiology of IRD through an integrative analysis of proteomic and biochemical data. A cohort of 203 patients with inherited retinal degeneration was genetically characterized by whole-exome sequencing (WES), followed by targeted profiling of lipid-related proteins, including apolipoprotein A1 (ApoA1), apolipoprotein E (ApoE) and lipoprotein lipase (LPL), together with the quantitative assessment of systemic lipid parameters. Integrative analyses identified a coordinated lipid–retinoid–photoreceptor metabolic network linking systemic lipid metabolism with photoreceptor-associated processes. Although conventional lipid measures largely remained within clinical reference ranges, their distributions revealed substantial biological variability. Lipid-associated network architecture defined distinct patterns of molecular organization and exhibited pronounced sex-dependent differences, with female patients displaying denser and more interconnected networks than male patients. Furthermore, the integration of genetic and protein expression demonstrated that variant pathogenicity is associated with the coordinated remodeling of lipid-related molecular states. Our findings identify lipid and retinoid metabolism as integral components of the molecular architecture underlying disease heterogeneity in IRD and reveal a lipid–retinoid metabolic network linking genetic variation to coordinated molecular remodeling. These results provide new insight into the molecular mechanisms contributing to photoreceptor-associated degeneration.

## 1. Introduction

Rare eye diseases constitute a diverse group of disorders characterized by a low prevalence in the human population and considerable clinical and biological heterogeneity. Inherited retinal degeneration (IRD) covers a genetically and clinically heterogeneous group of disorders characterized by progressive photoreceptor degeneration, ultimately leading to gradual vision loss and blindness [1]. The disease encompasses a broad spectrum of clinical phenotypes that differ in age of onset, rate of progression and the extent of rod and cone dysfunction. Despite substantial advances in genetic diagnostics and the identification of numerous IRD pathogenic variants, the ability to predict disease course based on genetic background only remains limited. Notably, pronounced clinical variability observed among patients carrying identical mutations suggests the existence of additional regulatory mechanisms modulating retinal responses to the primary molecular defect [2]. The traditional view of IRD pathogenesis has largely been based on a linear “gene-to-phenotype” model, in which pathogenic variants in genes essential for photoreceptor function are assumed to directly drive cellular dysfunction and retinal degeneration [3]. While this model has been fundamental to elucidating the molecular basis of the disease, it fails to account for the complexity of regulatory mechanisms between the genetic defect and the final cellular phenotype. Data indicate that the course of IRD is shaped by multilevel biological networks, in which cellular metabolism acts as an active modulator of disease progression [4]. Photoreceptors are among the most highly specialized and metabolically active neuronal cells, while their proper function is tightly dependent on lipid and retinoid metabolic process integrity. Lipids constitute a fundamental structural component of photoreceptor membranes, determining their fluidity, stability and capacity for continuous renewal [5]. At the same time, retinoid metabolism represents a core element of the visual cycle, ensuring efficient phototransduction and adaptation to fluctuating light conditions [6]. It is considered that disruptions within these pathways may lead to structural photoreceptor destabilization, impaired signaling and increased susceptibility to oxidative stress [7]. To date, alterations in lipid metabolism observed in the course of IRD have most often been linked to the secondary consequences of retinal degeneration, arising from photoreceptor loss and subsequent cellular and tissue remodeling [8]. However, such an approach limits the potential role of lipid dysregulation as an active contributor to disease pathophysiology. Growing evidence suggests that changes in lipid profiles or lipid metabolism-associated protein expression may occur early, prior to advanced photoreceptor loss, and may actively influence the rate and trajectory of disease progression [9]. Importantly, from a clinical point of view, lipid disturbances represent measurable, relatively stable parameters that are potentially amenable to therapeutic modulation. Consequently, they may serve as functional endophenotypic markers, enabling patient classification independently of the highly heterogeneous genetic background of IRD [10].

The integration of lipid parameters with the expression profiles of selected proteins and genetic information enables a multidimensional characterization of patients with IRD that extends beyond a conventional genotype-based description. The analysis of the relationships between proteins involved in lipid and retinoid metabolic pathways allows the identification of stable co-regulatory patterns that may reflect distinct functional states of photoreceptors as well as diverse trajectories of disease progression [11].

Here, we aimed to investigate the contribution of lipid and retinoid metabolism to the molecular heterogeneity of inherited retinal degeneration through the integrated analysis of genetic, proteomic and biochemical data. We hypothesized that lipid-associated pathways may constitute a coordinated molecular network linking genetic variation to disease-associated molecular remodeling. We investigated the organization of the lipid–retinoid network by integrating lipid-related proteins, systemic lipid parameters and sex-specific metabolic differences to identify molecular signatures associated with IRD. These analyses were designed to provide new insights into the molecular mechanisms that may underlie the contribution of lipid and retinoid metabolism to the molecular organization of inherited retinal degeneration.

## 2. Results

### 2.1. Marked Inter-Individual and Sex-Specific Variability in Systemic Lipid Metabolism in IRD

At the beginning, analyses focused on the characterization of selected biochemical parameters, including total cholesterol (TC), triglycerides (TG), high-density lipoprotein (HDL) and lactate dehydrogenase (LDH), across the entire study cohort. Despite substantial inter-individual variability across all assessed metabolic parameters, group-level mean values largely remained within established clinical reference ranges.

**Figure 1:**
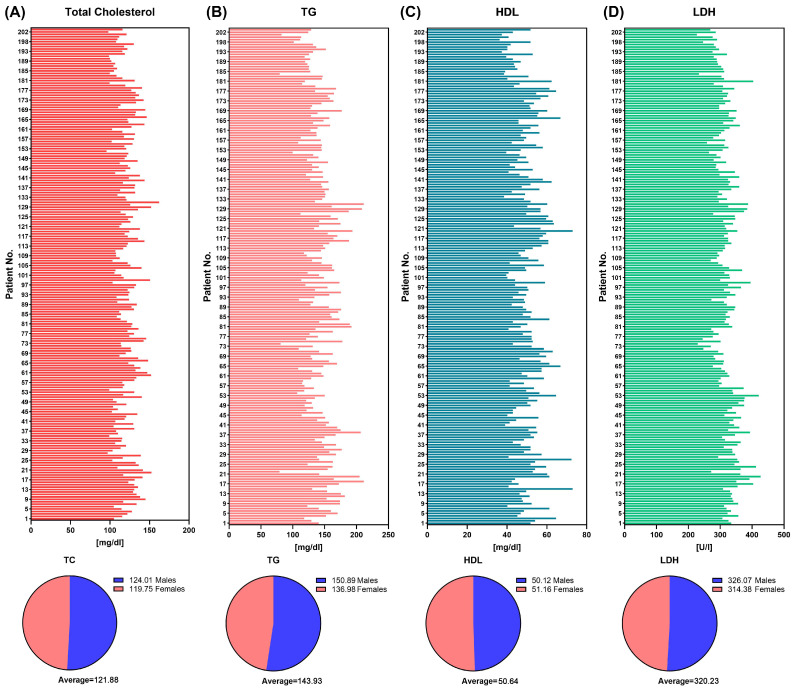
Systemic lipid phenotypes and metabolic heterogeneity in IRD patients. (**A**) Distribution of total cholesterol (TC), (**B**) triglycerides (TG), (**C**) high-density lipoprotein cholesterol (HDL) and (**D**) lactate dehydrogenase (LDH) activity across the IRD cohort with sex-dependent analysis.

In detail, the mean TC concentration was 121.88 mg/dL, with higher levels observed in men (124.01 mg/dL) compared to women (119.75 mg/dL). TC values were characterized by a broad range, from 95.60 mg/dL (mainly observed in women) to 162.07 mg/dL (mainly observed in men). Although none of the patients exceeded the clinical threshold for adult hypercholesterolaemia (<200 mg/dL), a subset—particularly among men—exhibited TC levels approaching the upper limit of norm (Figure 1A). Notably, the most pronounced and clinically relevant sex-dependent differences were observed for triglyceride levels. The mean TG concentration across the cohort was 143.93 mg/dL, with markedly higher values in men (150.89 mg/dL) than in women (136.98 mg/dL). TG concentrations ranged widely, from 79.12 mg/dL to 211.39 mg/dL. A substantial proportion of men exhibited TG levels exceeding the upper reference limit (<150 mg/dL), with several cases surpassing 200 mg/dL, indicative of moderate hypertriglyceridaemia. In contrast, elevated TG levels were less frequent and generally milder among women (Figure 1B). Analysis of the HDL fraction further revealed clear sex-dependent effects. Specifically, the mean HDL concentration in the overall cohort was 50.64 mg/dL, with higher levels observed in women (51.16 mg/dL) compared to men (50.12 mg/dL). HDL values ranged from 36.30 mg/dL to 72.96 mg/dL. Concentrations below the lower reference threshold (<40 mg/dL) were observed almost in men only, whereas women more frequently exhibited elevated HDL levels exceeding 60 mg/dL, with some cases approaching 70–73 mg/dL (Figure 1C). LDH activity also displayed marked inter-individual variability and a sex-dependent pattern. The mean LDH activity in the cohort was 320.23 U/L, with higher values in men (326.07 U/L) than in women (314.38 U/L). Observed LDH values ranged from 226.82 U/L to 426.67 U/L. In a subset of patients—predominantly men—LDH activity exceeded 400 U/L or approached the upper limit of the reference range (225–450 U/L), potentially reflecting increased catabolic activity, metabolic stress, or enhanced energy turnover. In women, LDH values were more frequently clustered within the mid-reference range and extreme elevations were rare (Figure 1D).

### 2.2. Distinct Lipid–Retinoid Protein Expression Phenotypes in IRD Patients

In the second stage, expression levels of five key proteins involved in lipid and retinoid metabolism (ApoA1, ApoE, LPL), rhodopsin (RHO) and aldehyde dehydrogenase 1 family, member A2 (ALDH1A2) were analyzed in all 203 IRD patients. Pronounced heterogeneity in expression profiles was observed between individuals and across proteins, indicating the presence of diverse metabolic phenotypes within the cohort.

**Figure 2:**
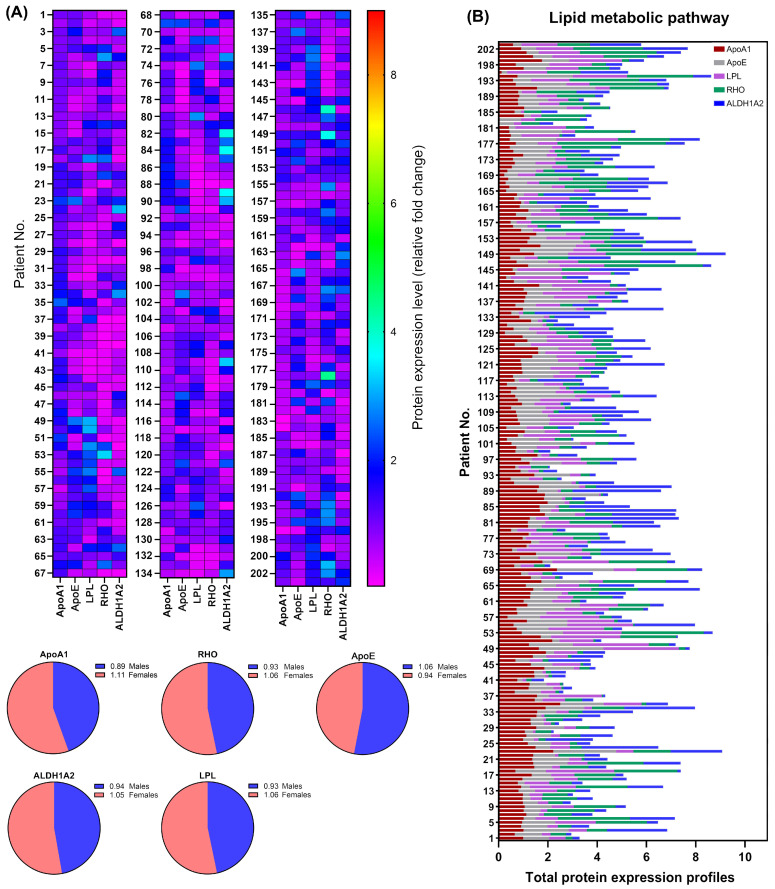
Protein expression profiles of lipid- and retinoid-associated pathways in IRD. (**A**) Heatmap representing expression profile of selected proteins with sex-dependent analysis; (**B**) total quantitative expression levels of ApoA1, ApoE, LPL, RHO and ALDH1A2 across the cohort.

ApoA1 expression was characterized by a broad dynamic range, from extremely low levels (LUC182) to very high relative expression reaching 2.49 (LUC35) and 2.36 (LUC69). In the majority of patients, ApoA1 expression fell within a moderate range (0.6–1.6). Sex-stratified analysis revealed that women more frequently displayed elevated ApoA1 levels (e.g., LUC23: 2.21; LUC35: 2.49; LUC69: 2.36), whereas men more often exhibited down-regulated expression (<0.7), including borderline or near-zero values (LUC182: 0.00; LUC183: 0.04). This pattern is consistent with a more favorable HDL-associated lipid phenotype in women. ApoE expression showed even greater variability, ranging from 0.08 (LUC75) to 2.78 (LUC101) and 2.75 (LUC165). High ApoE levels (>2.0) were observed in numerous patients of both sexes, but notably more frequently in men (e.g., LUC101, LUC165, LUC191). Conversely, several women exhibited very low ApoE expression (<0.3; e.g., LUC28: 0.33; LUC98: 0.16), underscoring substantial inter-individual differences in ApoE-dependent lipid transport. LPL expression similarly was characterized by an exceptionally wide range, from very low values (<0.2; e.g., LUC28: 0.10; LUC185: 0.12) to up-regulated levels exceeding 3.0 (LUC49: 3.06; LUC50: 3.08). High LPL expression (>2.0) was observed in both sexes and frequently co-occurred with elevated serum triglycerides, consistent with the compensatory activation of lipolytic pathways. At the same time, a substantial subset of patients exhibited low LPL expression (<0.5), suggestive of impaired triglyceride catabolism. Interestingly, rhodopsin (RHO) displayed the most extreme expression values across the entire panel. Expression ranged from near-zero levels (0.05–0.15; e.g., LUC37: 0.05; LUC40: 0.11) to very high values reaching 4.39 (LUC177), 3.99 (LUC146) and 3.70 (LUC149). Elevated RHO expression was more frequently observed in women and often coincided with increased LPL and ALDH1A2 expression, pointing to a coordinated photoreceptor–lipid metabolic axis. In men, RHO expression was more commonly moderate or low, although marked overexpression was also observed in selected cases (e.g., LUC53: 3.33; LUC194: 2.68). Finally, ALDH1A2 expression exhibited a distinctly asymmetric distribution, with numerous cases of very low expression (<0.1; e.g., LUC52: 0.06; LUC186: 0.04) alongside multiple instances of pronounced overexpression (>2.0). The highest ALDH1A2 levels were observed in patients LUC82 (3.81), LUC84 (3.67), LUC89 (3.59) and LUC109 (3.25). Elevated ALDH1A2 expression was more prevalent in women and frequently correlated with high RHO and LPL expression, consistent with intensified retinoid metabolism coupled to lipid dysregulation (Figure 2A).

To capture integrated metabolic activity, a composite protein expression score was calculated for each patient as the sum of relative expression level of proteins measured. These composite profiles varied widely, from low (<3 relative units) to very high (>8–9 units). The lowest scores were observed in patients with uniformly reduced expression across all proteins, whereas the highest values were driven by cumulative RHO, LPL and ALDH1A2 upregulation. Importantly, elevated composite expression typically reflected the selective activation of specific pathway components rather than uniform upregulation of the entire panel. This analysis revealed distinct molecular phenotypes characterized by low, intermediate and high global expression profiles. Women more frequently exhibited extreme high-expression profiles, driven primarily by elevated RHO and ALDH1A2 levels, whereas intermediate profiles predominated among men. Collectively, these findings reflect a heterogeneous, modular activation of the lipid–retinoid axis and support the existence of a continuous spectrum of metabolic endophenotypes in patients with inherited retinal degeneration (Figure 2B).

### 2.3. Sex-Specific Reorganization of Lipid–Retinoid Protein Interaction Networks

**Figure 3:**
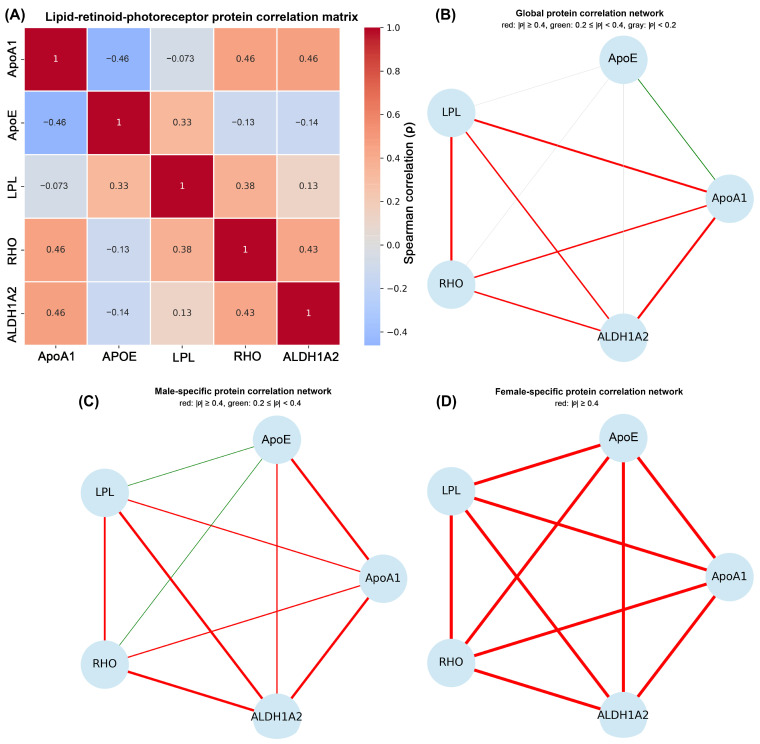
Correlation analysis and protein correlation networks of proteins involved in the lipid–retinoid axis.(**A**) Spearman correlation matrix illustrating the relationships among ApoA1, ApoE, LPL, RHO and ALDH1A2. (**B**) Global protein correlation network constructed based on Spearman correlation coefficients. (**C**,**D**) Sex-specific protein correlation networks illustrating differences in network organization and correlation strength between male and female patients.

Next, correlations among proteins involved in lipid or retinoid metabolism and photoreceptor function were examined using Spearman correlation matrices together with global and sex-stratified protein correlation networks. The Spearman correlation revealed several evident positive associations. The strongest correlations (|ρ| ≥ 0.4) were observed between RHO and ALDH1A2 (ρ = 0.43), ApoA1 and RHO (ρ = 0.46), ApoA1 and ALDH1A2 (ρ = 0.46), as well as between ApoA1 and ApoE (ρ = −0.46). A moderate positive correlation was detected between LPL and RHO (ρ = 0.38), while a weaker association was observed between ApoE and LPL (ρ = 0.33). Correlations involving ApoE-RHO, ApoE-ALDH1A2, ApoA1-LPL and LPL-ALDH1A2 were weak (|ρ| < 0.2) (Figure 3A). The global protein correlation network reflected this overall correlation structure, highlighting the strongest associations between ApoA1-RHO, ApoA1-ALDH1A2, ApoA1-ApoE and RHO-ALDH1A2. Moderate associations were noted for LPL-RHO and ApoE-LPL, while the other protein pairs exhibited weak correlations (Figure 3B). In turn, sex-stratified network analysis revealed marked differences in network organization and correlation strength. Specifically, for men, the protein correlation network was sparser and more heterogeneous, with strong positive associations maintained for ApoA1-ALDH1A2, ApoA1-RHO, ApoA1-ApoE, RHO-ALDH1A2 and RHO-LPL. Several ApoE- and LPL-related interactions, including ApoE-LPL and ApoE-RHO, were noted as moderate correlations (Figure 3C). In contrast, the female protein correlation network displayed substantially greater density and a higher number of strong positive associations. Most protein pairs fulfilled the criterion of |ρ| ≥ 0.4, encompassing ApoA1-ApoE, ApoA1-LPL, ApoA1-RHO, ApoA1-ALDH1A2, LPL-RHO, RHO-ALDH1A2, ApoE-RHO, ApoE-ALDH1A2 and ApoE-LPL, resulting in a highly interconnected network (Figure 3D).

### 2.4. Integration of Genetic and Proteomic Data Identifies Genotype-Specific Metabolic Endophenotypes

In the final stage, genetic data were integrated with protein expression profiles, revealing significant associations between variant pathogenicity status and expression levels of proteins involved in lipid metabolism, retinoid metabolism and photoreceptor function. Variant pathogenicity status was reflected in coordinated shifts in protein expression profiles across the analyzed protein set.

**Figure 4:**
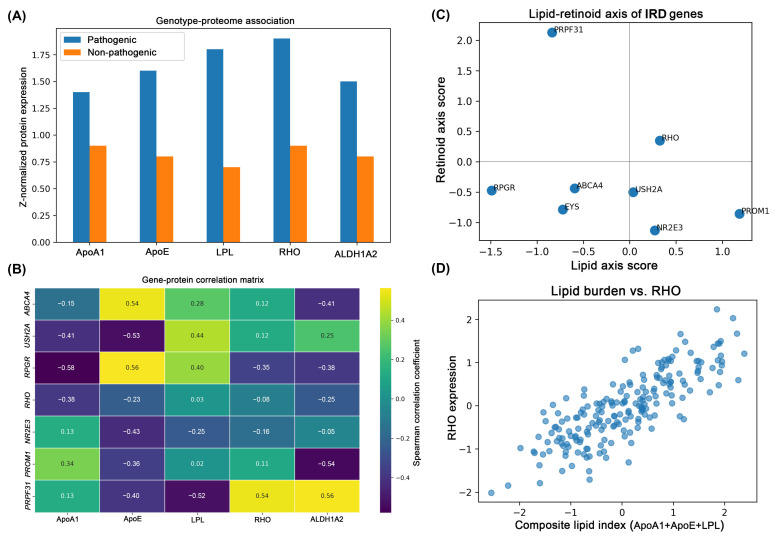
Integrated genetic–proteomic–metabolic framework in IRD patients.(**A**) Overview of non- and pathogenic gene variants mapped onto protein expression profiles; (**B**) multilayer network representation gene–protein correlation matrix; (**C**) lipid–retinoid axis of IRD-related genes; (**D**) composite lipid index (ApoA1, ApoE, LPL) versus RHO expression.

Protein expression varied according to variant pathogenicity classification, with higher mean expression of ApoA1, ApoE, LPL, RHO and ALDH1A2 observed in patients carrying pathogenic or likely pathogenic variants. The most pronounced differences were observed for LPL and RHO, for which mean normalized expression exceeded approximately 1.7 and 1.8 Z-score units, respectively, compared to values below 1.0 in the non-pathogenic group. ApoA1 and ALDH1A2 likewise showed a clear rightward shift in expression distribution in the pathogenic group, whereas differences for ApoE were more moderate but consistent (Figure 4A). A gene–protein correlation matrix further revealed distinct patterns of association between IRD-related genes and lipid–retinoid protein expression. The strongest positive Spearman correlations were observed between *PRPF31* and ALDH1A2 (ρ = 0.56), as well as RHO (ρ = 0.54). Moderate positive correlations were also identified between *RPGR* and ApoE (ρ = 0.56), as well as *RPGR* and LPL (ρ = 0.40). Negative correlations were observed for *RPGR*-ApoA1 (ρ = −0.58), *USH2A*-ApoE (ρ = −0.53) and *PROM1*-ALDH1A2 (ρ = −0.54). *ABCA4* showed a moderate positive correlation with ApoE (ρ = 0.54) and a negative correlation with ALDH1A2 (ρ = −0.41), whereas RHO exhibited generally weak correlations with the analyzed proteins (|ρ| < 0.3) (Figure 4B). The projection of IRD-associated genes into a two-dimensional space defined by lipid and retinoid axes revealed marked heterogeneity in molecular profiles. *PRPF31* occupied a position characterized by a high retinoid axis score and a negative lipid axis score. *PROM1* and *NR2E3* localized to regions of positive lipid and negative retinoid scores, whereas *RHO* assumed an intermediate position with positive values along both axes. *RPGR* and *EYS* clustered within regions of negative values for both components, while *ABCA4* and *USH2A* exhibited moderately negative lipid scores and slight shifts towards negative retinoid values (Figure 4C). Finally, the analysis of the association between the composite lipid index, defined as the summed expression of ApoA1, ApoE, LPL, and RHO expression revealed a clear positive linear association. Increasing values of the composite lipid index were accompanied by a progressive increase in RHO expression, with the highest RHO levels observed in patients exhibiting concurrent high expression of all three lipid-associated proteins (Figure 4D).

## 3. Discussion

In this study, we present an integrative, systems-level analysis of inherited retinal degeneration that reframes the disease beyond a mutation-centered paradigm and positions cellular metabolism as an active determinant of disease dynamics. By combining genetic background with proteomic profiles and systemic lipid phenotypes, we identify a functional molecular layer that bridges inherited defects with downstream cellular remodeling. Our findings support a model in which disturbances in lipid and retinoid metabolism are not only secondary consequences of photoreceptor degeneration, but instead constitute a central regulatory mechanism that modulates disease trajectory, network organization and even phenotypic heterogeneity in IRD.

Although we observed that standard clinical lipid parameters and LDH activity largely remained within reference ranges, their distribution across the cohort revealed pronounced metabolic heterogeneity. Importantly, this variability was structured and strongly influenced by the biological sex of the patients. Male individuals more frequently exhibited metabolic profiles characterized by elevated triglyceride levels, increased LDH activity and, simultaneously, reduced HDL concentrations. In turn, female patients tended to present more favorable lipid profiles with fewer extreme deviations. These findings indicate that biological sex may act as a significant modifier of the systemic metabolic landscape in IRD, even in the absence of overt dyslipidemia, and suggest that conventional clinical thresholds may mask biologically meaningful variation relevant to retinal disease mechanisms. Importantly, the sex-specific regulation of lipid metabolism has been documented extensively in human studies, with differences in lipid kinetics, triglycerides and HDL profiles observed between men and women across populations even when standard clinical lipid measures remain within normal ranges [12] and sex-dependent expression of lipid metabolism genes are reported in primary human cells [13]. Of note, sex hormones, particularly estrogens, are known to regulate lipid homeostasis, among others, through PPAR and LXR signaling pathways, providing one possible explanation for the observed sex-dependent metabolic profiles [14,15].

Beyond individual metabolic parameters, correlation-based analyses uncovered a coherent network of associations linking lipid transport, retinoid metabolism and photoreceptor-related proteins. The observed correlation structure was consistent with the presence of a coordinated lipid–retinoid–photoreceptor axis. In particular, the ApoA1- and ApoE-associated correlations suggest the existence of functionally distinct lipid transport pathways, consistent with previous studies describing the different roles of ApoA1/HDL and ApoE in systemic versus local lipid transport [16,17]. One mechanism appears aligned with HDL-mediated systemic lipid transport, as extensively documented in biochemical and clinical studies [16], whereas the other may reflect ApoE-dependent lipid trafficking with greater relevance to local retinal or glial metabolism. This interpretation is supported by experimental evidence demonstrating local ApoE expression and function in neural and retinal tissues [17]. This functional separation implies that different lipid pools may differentially influence photoreceptor homeostasis under degenerative stress. This concept is reflected in studies highlighting compartmentalized lipid metabolism and high lipid turnover in the outer retina [18,19].

Another aspect directly concerns rhodopsin, which is an integral membrane receptor coupled to a G protein. Its stability, transport and signaling efficiency critically depend on the lipid composition of the photoreceptor outer-segment membranes [20]. It is believed that cholesterol, phospholipids and polyunsaturated fatty acids modulate rhodopsin conformation, membrane packing and phototransduction efficiency [21]. Thus, disturbances in systemic lipid transport mediated by ApoA1, ApoE and LPL may indirectly influence photoreceptor homeostasis. This provides a biological explanation for the coordinated associations observed between lipid transport proteins and RHO expression in the present study. Also, the strong association between RHO and ALDH1A2 may reflect the coordinated regulation of retinoid metabolism and photoreceptor homeostasis [22]. In fact, ALDH1A2 catalyzes the synthesis of retinoic acid, a key regulator of gene transcription involved in retinal differentiation, maintenance and cellular stress response mechanisms [23,24]. Consequently, altered ALDH1A2 expression may reflect the adaptive remodeling of retinoid signaling involved in maintaining photoreceptor function during retinal degeneration.

Furthermore, lipoprotein lipase (LPL) occupied an intermediate position, linking systemic lipid metabolism with proteins directly associated with photoreceptor structure and function. This observation was consistent with experimental evidence demonstrating LPL expression and activity in the retina and its role in lipid uptake regulation in photoreceptors and retinal pigment epithelium [5,25]. Also, this pattern supports a role for LPL in regulating lipid availability at the interface between peripheral circulation and retinal cellular demands. This observation is consistent with the concept that disturbances in lipid metabolism contribute to metabolic stress responses implicated in inherited retinal degeneration, including oxidative stress and autophagy-related pathways [26,27]. Together, these findings contradict the entirely passive role of lipid metabolism in inherited retinal degeneration and instead support the concept of an adaptive metabolic response, consistent with growing evidence that metabolic reprogramming actively influences photoreceptor survival and vulnerability [26,28]. Moreover, photoreceptors are among the most metabolically active neurons in the human body, requiring a continuous supply of fatty acids to sustain outer-segment renewal, membrane biogenesis and mitochondrial energy production [26,29]. Altered LPL activity may therefore represent an adaptive response aimed at maintaining lipid supply during progressive retinal degeneration.

Protein correlation network analysis revealed a highly interconnected molecular network linking proteins involved in lipid transport, retinoid metabolism and photoreceptor function. The topology of this network suggests that metabolic remodeling in IRD follows structured regulatory principles rather than stochastic dysregulation [11,30,31]. Such organization is consistent with a systems-level response in which metabolic pathways actively integrate genetic signals with cellular stress-adaptation mechanisms, a effect observed across proteomic and network studies of inherited retinal degeneration [31,32].

More important, the architecture of these networks differed substantially between sexes. This pattern is supported by previous studies reporting sex-dependent differences in retinal degeneration and disease progression [33,34]. In male patients, the correlation network displayed higher heterogeneity, with a mixture of strong and moderate associations, indicative of a more flexible or plastic metabolic configuration. This observation is also reflected by evidence that metabolic and degenerative trajectories differ by sex in IRD models [33]. In contrast, female patients exhibited a denser network dominated by strong positive correlations across nearly all proteins. Such results suggest a more tightly coordinated but potentially less adaptable regulatory system, mirroring observations that female IRD models show accelerated retinal degeneration compared to males and altered metabolic vulnerability [33,35]. Together, these sex-specific network configurations point to divergent metabolic strategies in response to retinal degeneration and may contribute to differences in disease trajectories that are not captured by genetic variation alone, in line with growing evidence that sex acts as a critical biological modifier-factor of IRD progression and trait expression [34]. One possible explanation for the denser protein correlation network observed in women is the well-established role of estrogens in regulating lipid metabolism and retinal homeostasis [36]. Estrogens influence HDL metabolism, ApoA1 expression and cholesterol homeostasis through multiple nuclear receptor pathways, including PPAR and LXR signaling [14,37]. These mechanisms may facilitate tighter metabolic coordination under retinal stress and could contribute to the more interconnected lipid–retinoid network observed in female patients.

In summary, our findings support a hypothetical model in which genetic defects initiate chronic metabolic stress that progressively remodels lipid transport, retinoid metabolism and photoreceptor homeostasis. In this model, ApoA1, ApoE and LPL contribute to lipid transport and availability while ALDH1A2 modulates retinoid signaling. In turn, rhodopsin represents a downstream molecular integrator of these pathways within photoreceptors. Differences in this integrated network may ultimately contribute to the marked molecular signature observed among patients with inherited retinal degeneration.

### Study Limitations

The present study has several limitations that should be considered when interpreting the findings. The study cohort did not include healthy subjects as controls. Consequently, the identified lipid–retinoid signatures cannot be interpreted as disease-specific molecular alterations, nor do they permit direct comparisons between physiological and disease-associated metabolic states. However, the primary objective of this study was to investigate genotype-associated molecular heterogeneity within a genetically and clinically well-characterized cohort of patients with inherited retinal degeneration, instead of diagnostic biomarker identification or molecular differences between affected and unaffected patient characterization. Also, our systems-level approach enabled the identification of molecular endophenotypes associated with distinct genetic backgrounds, addressing a biological question that differs fundamentally from disease-versus-control investigations. Despite promising findings, further studies, including a healthy control group, together with independent IRD populations, are highly requested to validate the disease specificity, diagnostic relevance and broader clinical applicability of the lipid–retinoid molecular signatures identified in this study.

## 4. Materials and Methods

### 4.1. Study Cohort Characteristics and Ethical Considerations

The study included 203 patients with clinically confirmed inherited retinal degeneration (IRD), whose diagnoses were also characterized by comprehensive molecular analysis using whole-exome sequencing (WES). The cohort comprised patients with retinitis pigmentosa (isolated or syndromic) mainly, together with individuals affected by other genetically characterized IRD phenotypes, especially Usher syndrome, cone–rod dystrophy, Leber congenital amaurosis, Bardet–Biedl syndrome and other rare IRDs (Table 1). Participants were recruited and classified at the Department of General Ophthalmology, Medical University of Lublin, between July 2023 and October 2024. The study protocol was approved by the local Bioethics Committee (approval no. KE-0254/260/12/2022, 15 December 2022) and conducted in accordance with the Declaration of Helsinki. Written informed consent was obtained from all participants prior to enrollment.

### 4.2. Collection and Processing of Biological Material

Peripheral blood samples were collected into EDTA-containing tubes. Following fractionation, a part of the samples was centrifuged to obtain serum, while the remaining material was prepared for molecular analyses or stored at −80°C.The material for genetic diagnostics was transported on dry ice to the CeGaT laboratory (Tübingen, Germany). After completion of the analyses, residual blood samples, DNA and protein lysates were disposed of in accordance with applicable biosafety procedures.

### 4.3. Genetic Analysis

Molecular diagnostics were performed using whole-exome sequencing (WES). Library preparation and sequencing were carried out on the Illumina NovaSeq 6000 platform (Illumina Inc., San Diego, CA, USA). Bioinformatic analysis focused on the identification of variants in genes associated with inherited retinal dystrophies.

### 4.4. Determination of Global Lipid Markers in Serum

Serum concentrations of total cholesterol (TC), triglycerides (TG), high-density lipoprotein (HDL) cholesterol and lactate dehydrogenase (LDH) activity were measured using enzymatic assays with commercially available diagnostic kits (Cormay Diagnostics: Liquick Cor-CHOL, Liquick Cor-TG, HDL Direct, Liquick Cor-LDH; Lublin, Poland) according to the manufacturer’s instructions.

### 4.5. Protein Profiling by Western Blot

Peripheral blood mononuclear cells (PBMCs) were isolated using density gradient centrifugation. Whole blood was diluted 1:1 with PBS, layered onto a lymphocyte separation medium (density 1.077 g/mL) and centrifuged (700× *g*, 20 min, room temperature). The PBMC layer was collected, washed with PBS and resuspended in 2% SDS containing protease inhibitors. Cell lysates were mechanically homogenized using zirconium beads (1.0–1.2 mm) in a VWR Bead Mill Max homogenizer (VWR International LLC, Radnor, PA, USA), applying five homogenization cycles of 45 s each (5.0 m/s). Protein concentration was determined using the BCA assay. For SDS-PAGE, 10 µg of protein per sample was loaded, followed by transfer onto PVDF membranes according to [38]. Membranes were blocked for 1 h in 1–3% BSA and then incubated overnight at 4°C with the following primary antibodies (all at 1:1000 dilution): anti-β-actin (Cat# 4967, RRID: AB_330288, Cell Signaling Technology, Danvers, MA, USA), anti-ApoA1 (Cat# 3350, RRID: AB_2227261, Cell Signaling Technology), anti-ApoE (Cat# 13366, RRID: AB_2798191, Cell Signaling Technology), anti-LPL (Cat# MA5-32688, RRID: AB_2809965, Thermo Fisher Scientific, Waltham, MA, USA), anti-rhodopsin (RHO) (Cat# MA1-722, RRID: AB_325050, Thermo Fisher Scientific, Waltham, MA, USA) and anti-ALDH1A2 (Cat# PA5-50112, RRID: AB_2635565, Thermo Fisher Scientific, Waltham, MA, USA). Following incubation with primary antibodies, membranes were incubated for 1 h at room temperature with horseradish peroxidase (HRP)-conjugated secondary antibodies: anti-mouse (1:40,000, Cat# A9044, Sigma-Aldrich, St. Louis, MO, USA) or anti-rabbit (1:40,000, Cat# A0545, Sigma-Aldrich, St. Louis, MO, USA). Signal detection was performed by chemiluminescence using the Westar Supernova ECL detection kit (Cyanagen, Bologna, Italy).

## 5. Conclusions

Our findings identify lipid and retinoid metabolism as integral components of the molecular network underlying disease heterogeneity in inherited retinal degeneration. Metabolic alterations emerge as coordinated, sex-dependent networks associated with distinct patterns of molecular organization. Integration of genetic, proteomic and biochemical data supports the existence of a lipid–retinoid metabolic network linking genetic variation to molecular heterogeneity. Taken together, these findings provide a systems-level perspective on the biological processes underlying inherited retinal degeneration and highlight lipid-associated pathways as potential contributors to disease progression.

## Figures and Tables

**Table 1:** Demographic, clinical and genetic characteristics of the study cohort.

Characteristic	Overall Cohort (*n* = 203)
**Demographic characteristics**	
Age (years), mean ± SD	43.1 ± 15.1
Age (years), median (IQR)	43 (33–54)
Age range (years)	10–81
Female, *n* (%)	102 (52.2%)
Male, *n* (%)	101 (49.8%)
**Clinical characteristics**	
Retinitis pigmentosa	136 (67.0%)
Usher syndrome	21 (10.3%)
Cone-rod dystrophy	12 (5.9%)
Leber congenital amaurosis	2 (1.0%)
Bardet–Biedl syndrome	4 (2.0%)
Other inherited retinal degenerations	2 (1.0%)
Additional phenotypes	Remaining patients
**Genetic characteristics**	
Whole-exome sequencing performed	203 (100%)
Patients with a confirmed molecular diagnosis	166 (81.8%)
Number of IRD genes represented	73
Most frequently affected genes	*USH2A*, *ABCA4*, *RPGR*, *EYS*, *NR2E3*, *PROM1*, *RHO*, *PRPF31*

Abbreviations: IQR, interquartile range; IRD, inherited retinal degeneration.

## Data Availability

The data presented in this study are available from the corresponding author upon reasonable request. The data are not publicly available due to privacy and ethical restrictions associated with patient-derived clinical and genetic information.

## References

[1] O’Neal T.B., Tripathy K., Luther E.E. (2025). Retinitis Pigmentosa. StatPearls.

[2] Daich Varela M., Romo-Aguas J.C., Guarascio R., Ziaka K., Aguila M., Hau K.L., Li Y., Chen R., Kalitzeos A., Robson A.G. (2025). RHO-Associated Retinitis Pigmentosa: Genetics, Phenotype, Natural History, Functional Assays, and Animal Model—In Preparation for Clinical Trials. Investig. Ophthalmol. Vis. Sci..

[3] Hamel C. (2006). Retinitis pigmentosa. Orphanet J. Rare Dis..

[4] Esteban-Medina M., Loucera C., Rian K., Velasco S., Olivares-González L., Rodrigo R., Dopazo J., Peña-Chilet M. (2024). The mechanistic functional landscape of retinitis pigmentosa: A machine learning-driven approach to therapeutic target discovery. J. Transl. Med..

[5] Lewandowski D., Sander C.L., Tworak A., Gao F., Xu Q., Skowronska-Krawczyk D. (2022). Dynamic lipid turnover in photoreceptors and retinal pigment epithelium throughout life. Prog. Retin. Eye Res..

[6] Tang P.H., Kono M., Koutalos Y., Ablonczy Z., Crouch R.K. (2013). New insights into retinoid metabolism and cycling within the retina. Prog. Retin. Eye Res..

[7] Pan W.W., Wubben T.J., Besirli C.G. (2021). Photoreceptor metabolic reprogramming: Current understanding and therapeutic implications. Commun. Biol..

[8] Liu W., Liu S., Li P., Yao K. (2022). Retinitis Pigmentosa: Progress in Molecular Pathology and Biotherapeutical Strategies. Int. J. Mol. Sci..

[9] Fu Z., Chen C.T., Cagnone G., Heckel E., Sun Y., Cakir B., Tomita Y., Huang S., Li Q., Britton W. (2019). Dyslipidemia in retinal metabolic disorders. EMBO Mol. Med..

[10] Wang W.C., Huang C.H., Chung H.H., Chen P.L., Hu F.R., Yang C.H., Yang C.M., Lin C.W., Hsu C.C., Chen T.C. (2024). Metabolomics facilitates differential diagnosis in common inherited retinal degenerations by exploring their profiles of serum metabolites. Nat. Commun..

[11] Montaser A.B., Gao F., Peters D., Vainionpää K., Zhibin N., Skowronska-Krawczyk D., Figeys D., Palczewski K., Leinonen H. (2024). Retinal Proteome Profiling of Inherited Retinal Degeneration Across Three Different Mouse Models Suggests Common Drug Targets in Retinitis Pigmentosa. Mol. Cell. Proteom..

[12] Wang X., Magkos F., Mittendorfer B. (2011). Sex differences in lipid and lipoprotein metabolism: It’s not just about sex hormones. J. Clin. Endocrinol. Metab..

[13] Seidemann L., Lippold C.P., Rohm C.M., Eckel J.C., Schicht G., Matz-Soja M., Berg T., Seehofer D., Damm G. (2024). Sex hormones differently regulate lipid metabolism genes in primary human hepatocytes. BMC Endocr. Disord..

[14] Yoon M. (2009). The role of PPARalpha in lipid metabolism and obesity: Focusing on the effects of estrogen on PPARalpha actions. Pharmacol. Res..

[15] Li A.C., Glass C.K. (2004). PPAR- and LXR-dependent pathways controlling lipid metabolism and the development of atherosclerosis. J. Lipid Res..

[16] Kontush A., Chapman M.J. (2006). Functionally defective high-density lipoprotein: A new therapeutic target at the crossroads of dyslipidemia, inflammation, and atherosclerosis. Pharmacol. Rev..

[17] Mahley R.W., Huang Y. (2012). Apolipoprotein e sets the stage: Response to injury triggers neuropathology. Neuron.

[18] Saari J.C. (2012). Vitamin A metabolism in rod and cone visual cycles. Annu. Rev. Nutr..

[19] Adijanto J., Du J., Moffat C., Seifert E.L., Hurle J.B., Philp N.J. (2014). The retinal pigment epithelium utilizes fatty acids for ketogenesis. J. Biol. Chem..

[20] Gulati S., Palczewski K. (2023). Structural view of G protein-coupled receptor signaling in the retinal rod outer segment. Trends Biochem. Sci..

[21] Jastrzebska B., Debinski A., Filipek S., Palczewski K. (2011). Role of membrane integrity on G protein-coupled receptors: Rhodopsin stability and function. Prog. Lipid Res..

[22] Engfer Z.J., Palczewski K., Duester G., Ghyselinck N.B. (2025). Chapter Nine—The multifaceted roles of retinoids in eye development, vision, and retinal degenerative diseases. Current Topics in Developmental Biology.

[23] Gudas L.J. (2022). Retinoid metabolism: New insights. J. Mol. Endocrinol..

[24] Thompson B., Katsanis N., Apostolopoulos N., Thompson D.C., Nebert D.W., Vasiliou V. (2019). Genetics and functions of the retinoic acid pathway, with special emphasis on the eye. Hum. Genom..

[25] Fliesler S.J., Bretillon L. (2010). The ins and outs of cholesterol in the vertebrate retina. J. Lipid Res..

[26] Aït-Ali N., Fridlich R., Millet-Puel G., Clérin E., Delalande F., Jaillard C., Blond F., Perrocheau L., Reichman S., Byrne L.C. (2015). Rod-derived cone viability factor promotes cone survival by stimulating aerobic glycolysis. Cell.

[27] Trachsel-Moncho L., Benlloch-Navarro S., Fernández-Carbonell Á., Ramírez-Lamelas D.T., Olivar T., Silvestre D., Poch E., Miranda M. (2018). Oxidative stress and autophagy-related changes during retinal degeneration and development. Cell Death Dis..

[28] Chen C.T., Shao Z., Fu Z. (2022). Dysfunctional peroxisomal lipid metabolisms and their ocular manifestations. Front. Cell. Dev. Biol..

[29] Hurley J.B., Lindsay K.J., Du J. (2015). Glucose, lactate, and shuttling of metabolites in vertebrate retinas. J. Neurosci. Res..

[30] Yoon S.B., Ma Y.C., Venkat A., Liu C.A., Zheng J.J. (2022). Applying Protein-Protein Interactions and Complex Networks to Identify Novel Genes in Retinitis Pigmentosa Pathogenesis. Int. J. Mol. Sci..

[31] Yang M., Qiu R., Jin X., Yao S., Wang W., Liu J., Liu G., Han J., Lei B. (2024). Proteomics identifies multiple retinitis pigmentosa associated proteins involved in retinal degeneration in a mouse model bearing a Pde6b mutation. Sci. Rep..

[32] Stehle I.F., Imventarza J.A., Woerz F., Hoffmann F., Boldt K., Beyer T., Quinn P.M., Ueffing M. (2024). Human CRB1 and CRB2 form homo- and heteromeric protein complexes in the retina. Life Sci. Alliance.

[33] Li B., Gografe S., Munchow A., Lopez-Toledano M., Pan Z.H., Shen W. (2019). Sex-related differences in the progressive retinal degeneration of the rd10 mouse. Exp. Eye Res..

[34] Hughes M.J., Lamey T., Schiff E.R., Lin S., McLaren T., Thompson J., Stephenson K.A.J., Sergouniotis P., Pontikos N., Varela M.D. (2025). Sex Distributions in the Most Frequent Autosomal Genetic Causes of Retinitis Pigmentosa. Investig. Ophthalmol. Vis. Sci..

[35] Liu H., Zhang X., Wang Q., Li B., Bian B., Liu Y. (2025). A Comprehensive Analysis of Sex-Biased Gene Expression in the Aging Human Retina Through a Combination of Single-Cell and Bulk RNA Sequencing. Investig. Ophthalmol. Vis. Sci..

[36] Nuzzi R., Scalabrin S., Becco A., Panzica G. (2018). Gonadal Hormones and Retinal Disorders: A Review. Front. Endocrinol..

[37] Lamon-Fava S., Micherone D. (2004). Regulation of apoA-I gene expression: Mechanism of action of estrogen and genistein. J. Lipid Res..

[38] Koszła O., Sołek P., Jóźwiak K. (2025). Co-treatment Strategy Supports Neuroprotection by Intersecting p62-Keap1-NRF2 and Autophagy Signaling Pathways in the Cellular Model of Parkinson’s Disease. Cell. Mol. Neurobiol..

